# The publication trend of neuropathic pain in the world and China: a 20–years bibliometric analysis

**DOI:** 10.1186/s10194-018-0941-4

**Published:** 2018-11-15

**Authors:** Jishi Ye, Huang Ding, Juan Ren, Zhongyuan Xia

**Affiliations:** 1 Department of Anesthesiology, Renmin Hospital of Wuhan University, No. 99 Zhang Road, Wuhan, 430060 Hubei People’s Republic of China; 2 Department of Respiratory, Wuhan general hospital of the People’s liberation Army, Wuhan, 430060 Hubei People’s Republic of China

**Keywords:** Bibliometric analysis, China, PubMed, Neuropathic pain, Web of science

## Abstract

**Background:**

There has been tremendous change on neuropathic pain research in the past 20 years in China and around the world. We analyzed the global trend of neuropathic pain research and compared China’s quantity and quality of neuropathic pain-related publications with other developed countries.

**Methods:**

Using terms “neuropathic pain”, we retrieved related publications from the Web of Science (WOS) database and PubMed database. From different aspects, such as the number of papers, total citations, average citations per item, H-index, research types, orientation, institutions, journals and funding, global neuropathic pain publications were classified and analyzed.

**Results:**

From 1998 to 2017, 21,733 articles regarding neuropathic pain research were published worldwide. Of these, 9.394% were contributed by authors from Chinese institutions, which followed USA and ranked second. However, the quality indicators of publications, including total citations, average citations per item and H-index, were relatively low in China. High contribution journals and the 10 most-cited articles on neuropathic pain in world and China were also listed, which also can reflect the quality of neuropathic pain. Based on National Natural Science Foundation of China (NSFC), basic research was the main articles type, accounting for 32.91% of China’s neuropathic pain research.

**Conclusion:**

Global neuropathic pain research increased rapidly during the 1998 to 2017 period. The USA was still the leader of neuropathic research. Although China had made great achievements, there was a significant gap in the high-quality studies between China and other leading countries.

## Introduction

Neuropathic pain, a pain syndrome caused by a lesion or disease of the somatosensory system, is a major public health concern and becoming the global burden [1–3]. Epidemiological research showed that the prevalence of neuropathic pain is likely to lie between 6.9% and 10% and cost increases year by year [4, 5]. According to site of major pathology, the classification of neuropathic pain also includes pathology, peripheral, spinal and brain [6]. Yet, few studies, until now, have uncovered the precise mechanism and a therapeutic approach. In the past two decades, innumerable research and money focused on this field to make clear the underling mechanism of neuropathic pain and therapeutic target. Meanwhile, many related research results were published in all kinds of journals in the form of articles [7–9] . Along with the tremendous economic growth, China’ scientific strength grows rapidly and achieves great accomplishments in neuropathic pain field. However, current studies have not shown the global and China’s development trend regarding neuropathic pain yet.

Bibliometrics is concerned with the analysis of research based on citation counts and patterns. This method can be used to evaluate the influence of an individual research output, such as a journal article, or a collection of research outputs, such as all works by a particular author, research group or institution. Different fields, such as anesthesiology [10], respiratory medicine [11], urology [12], and cancer [13] have performed this method to measure and rank research output both within institutions and on a national or international level.

To evaluate the quantity and quality of global neuropathic pain research, we utilized the bibliometrics analysis to analyze the research progress and growing trend in this field during two decades 2008–2017. At the same time, we also compared the neuropathic pain publications records from Chinese institutions and other developed countries to make clear the China’s contribution to neuropathic pain research and a gap in the quality of publications between China and other developed countries.

## Methods

### Data sources

This study was conducted based on previous similar publications. All data were acquired on September 20, 2018. Consider that these data were downloaded from the public databases and there existed no ethical questions about them, we did not apply the ethical approval. Neuropathic pain-related articles published between 1998 and 2017 were retrieved from PubMed database and the Web of Science (WOS) online database, which included the Science Citation Index Expanded (SCIE), Social Sciences Citation Index (SSCIE) and Arts & Humanities Citation Index(A&HCI). The journal impact factors (IF) came from Journal Citation Reports 2015 database. Foundation data from China were derived from the Latest scientific fund results query system (http://www.letpub.com.cn/index.php?page=grant). In addition, research types, including randomized controlled trials (RCTs), clinical trials, and case reports, were retrieved from the PubMed database.

### Search strategy

In WOS, the search terms were: Theme = (neuropathic pain) AND publishing year = (1998–2017). Literature type included article, review, meeting abstract, proceedings paper and letter. In PubMed, the search terms were: Mesh = (neuropathic pain) AND publication date (1998/01/01–2017/12/31). Literature type included basic research, randomized controlled trials, clinical trials and case reports. To search for basic research, we identified the species as “other Animals.” Publication quality was assessed by using total citations frequency, average citations per item and H-index. Literature quantity and publication trend were analyzed by total publications, research types, research orientations, research organization, author’s contribution, journal and funding support.

### Statistical analysis

Descriptive statistical analyses were mainly used. Trends during the two decades studied were analyzed using linear regression by SPSS 12.0 (SPSS Inc., Chicago, IL, USA). *P* values less than 0.05 were considered significant.

## Results

### Neuropathic pain related articles in world and in China

**Fig. 1:**
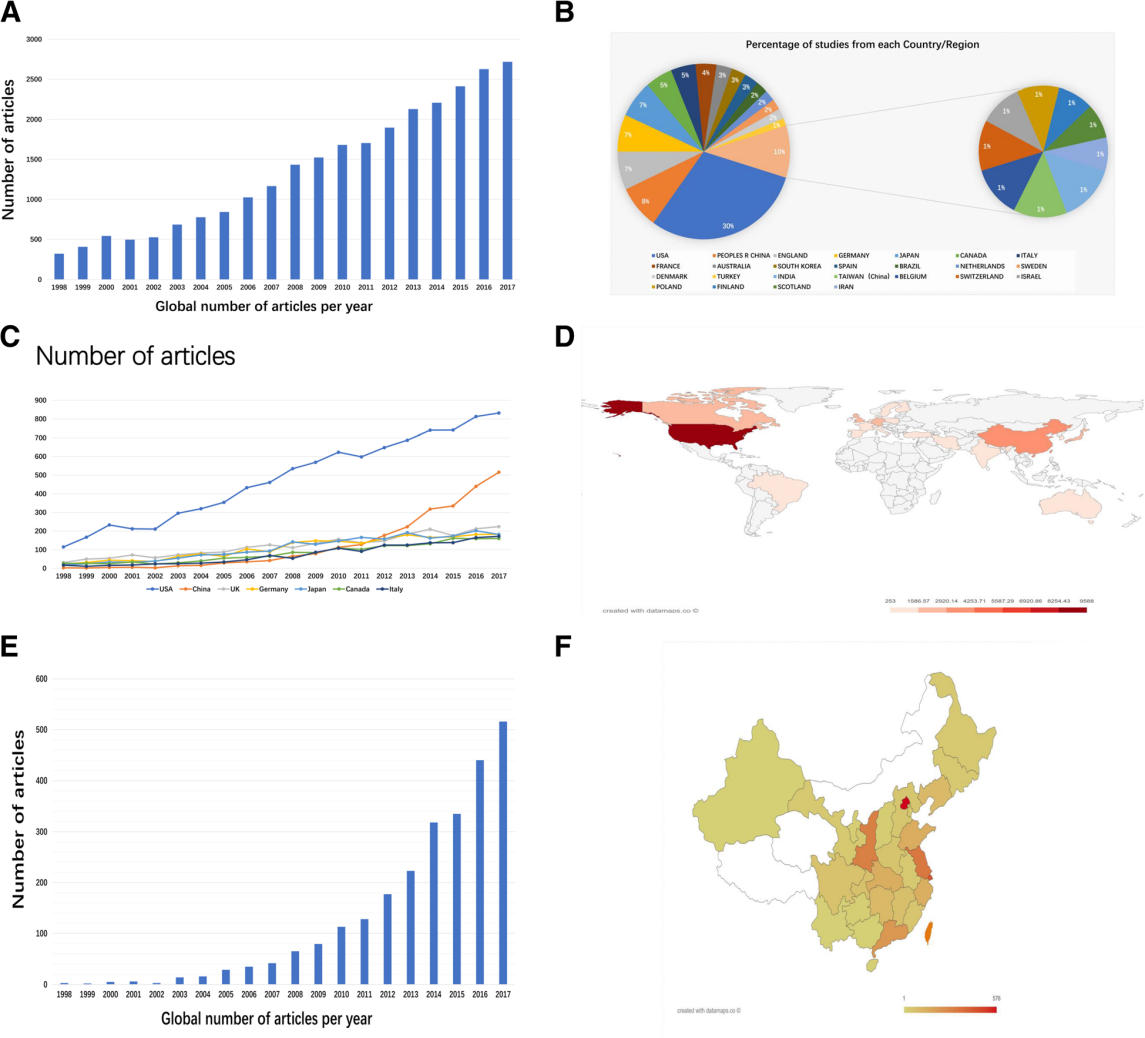
Neuropathic pain related articles in world and in China (**a**) The global number of neuropathic pain publications. The blue bars indicate the quantity of neuropathic pain articles. (**b**) Percentage of studies from each Country/Region. (**c**) The time curve of neuropathic pain articles from top 7 countries. (**d**) The heat map showing the distribution of neuropathic pain publications in world. (**e**) The annual number of neuropathic pain articles in China. (**f**) The heat map showing the distribution of neuropathic pain publications in China

Based on search criteria, a total of 21,733 articles were published in world from 1998 to2017 in WOS database. The global number of publications regarding neuropathic pain displayed a positive growth trend (R^2^ = 0.9776, *P* < 0.01), from 320 in 1998 to 2723 in 2017. Nearly 99 countries/regions participated in global neuropathic pain research. Among them, the USA published the largest number of neuropathic pain articles (9589, 35.341%), followed by China (2549, 9.394%), England (2254, 8.307%), Germany (2183, 8.046%), and Japan (2183, 8.046%). Like global publication trend, the annual publications of neuropathic pain in these countries all showed a remarkably positive trend (Fig. 1a-d).

In China, a total of 2549 articles on neuropathic pain were published between 1998 to 2017 and showed a strongly growth as well (R^2^ = 0.7853, *P* < 0.01). Among all provinces, Beijing produced the most papers in the past two decades (Fig. 1e-f).

### Citations and h-index analysis in world and China

**Fig. 2:**
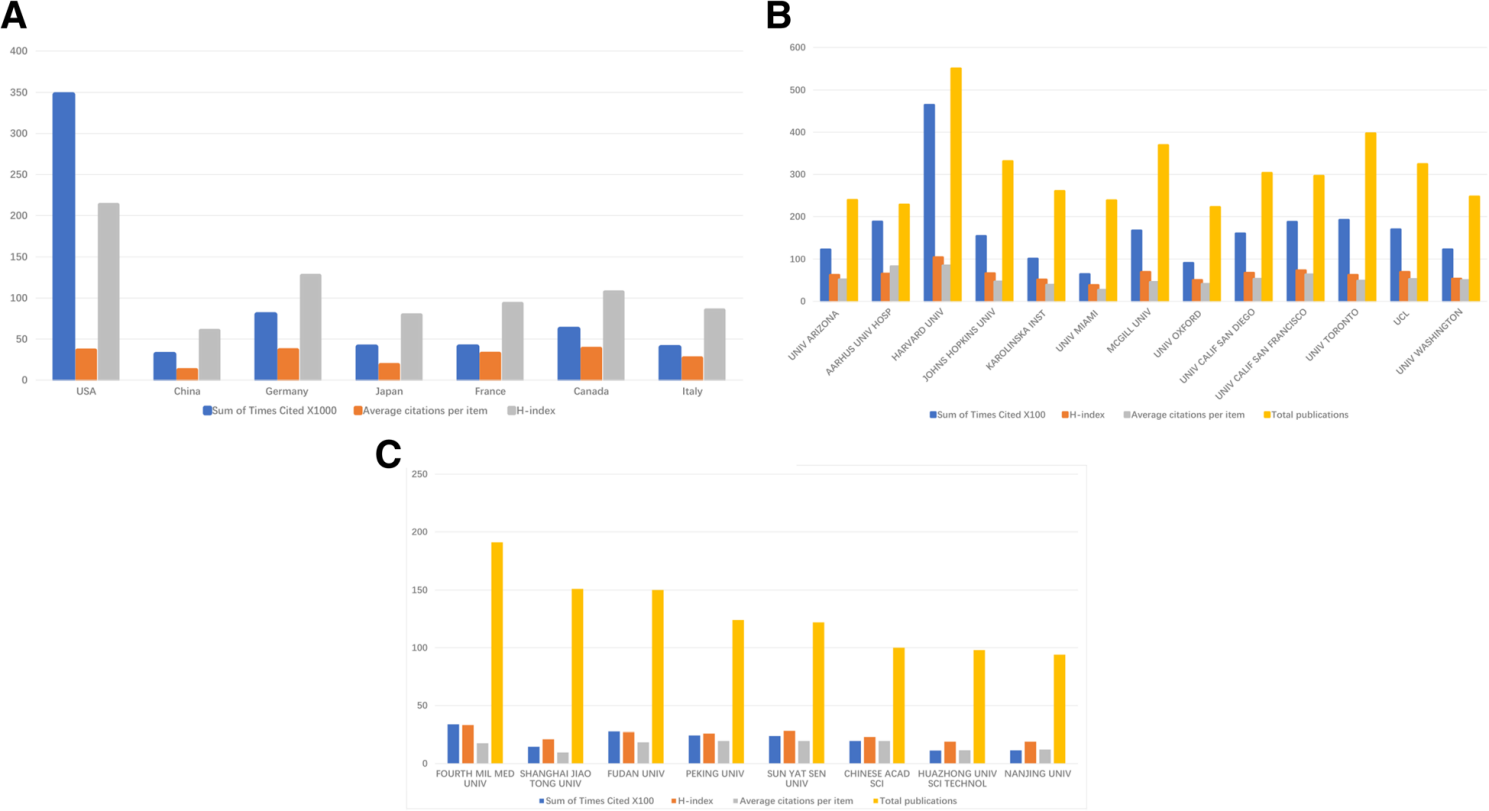
Citations and h-index analysis in world and China (**a**) The total citations, average citations per paper and H-index for neuropathic pain articles from top countries. (**b**) The total citations, average citations per paper and H-index for neuropathic pain articles in the global institutions. (**c**) The total citations, average citations per paper and H-index for neuropathic pain articles in Chinese institutions

According to the WOS database, neuropathic pain papers from the USA possessed most frequently citations, with 347,125 total citations and average 36.2 citations per item during the past 20 years. In terms of the H-index, the index of the USA was 213, which was higher than that of any other country or region (Fig. 2a). Despite the number of articles ranked the second, the total citations and H-index of papers published in China (31,148 total citations, 12.22 citations per item and 60 H-index) were behind other developed countries.

One thousand three hundred sixty eight institutions from different nations or regions contributed to neuropathic pain research between 1998 and 2017. The top 13 most contributing institutions in the world are listed in Fig. 2b. Among these institutions, 9 were from the USA, University of Oxford was in UK, University of Toronto was from Canada, Karolinska institution was from Sweden and Aarhus University Hospital was in Denmark. The institution with the largest amount of neuropathic pain articles was Harvard University, which produced 550 papers and had 46,432 total citations and 104 h-index. In China, Fourth Military Medical University ranked first, which published 191 papers with 3361 total citations and 33 H-index (Fig. 2c). Obviously, there is a huge gap between the world’s top institutions and Chinese institutions regarding on neuropathic pain. And these differences should not be ignored.

### The difference of research categories and article types in world and China

**Fig. 3:**
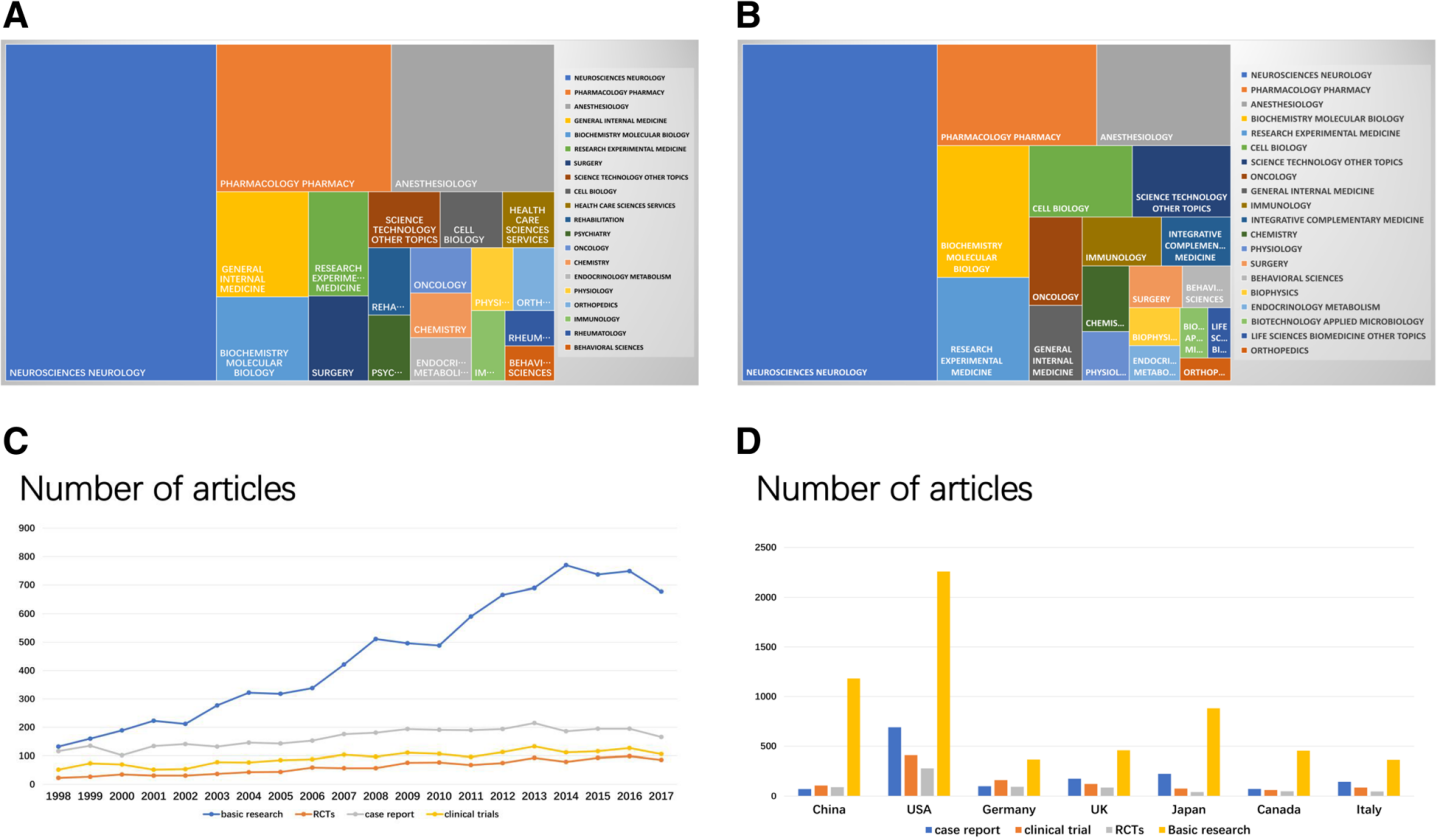
The difference of research categories and article types in world and China (**a**) The research categories on neuropathic pain in world. (**b**) The research categories on neuropathic pain in China. (**c**) The article types analysis regarding neuropathic pain in the past two decades worldwide. (**d**) The article types analysis regarding neuropathic pain from top countries in the past two decades

In world, there were 127 research categories on neuropathic pain articles, among which neuroscience neurology (13,425, 49.491%), pharmacology (4883,18.001%), anesthesiology (4550, 16.774%), general internal medicine (1831, 6.75%), and biochemistry molecular biology (1466,5.404%) were the most common areas (Fig. 3a). In this regard, the research categories of Chinese articles on neuropathic pain are similar with the global articles (Fig. 3b).

In the past 20 years, global researchers mainly focused on basic research with 8967 papers, accounting for 32.91% of the total neuropathic papers in PubMed database (Fig. 3c). And the growth rate keeps increase. Additionally, 3258 case reports (11.94%), 1752 clinical trials (6.42%), and 1190 RCTs (4.36%) were published in the neuropathic pain field. The amount of papers for each research types from the USA far exceed other countries. Although China ranked second, the gap is still considerable (Fig. 3b).

### High contribution journals in neuropathic pain field and funds in China

**Fig. 4:**
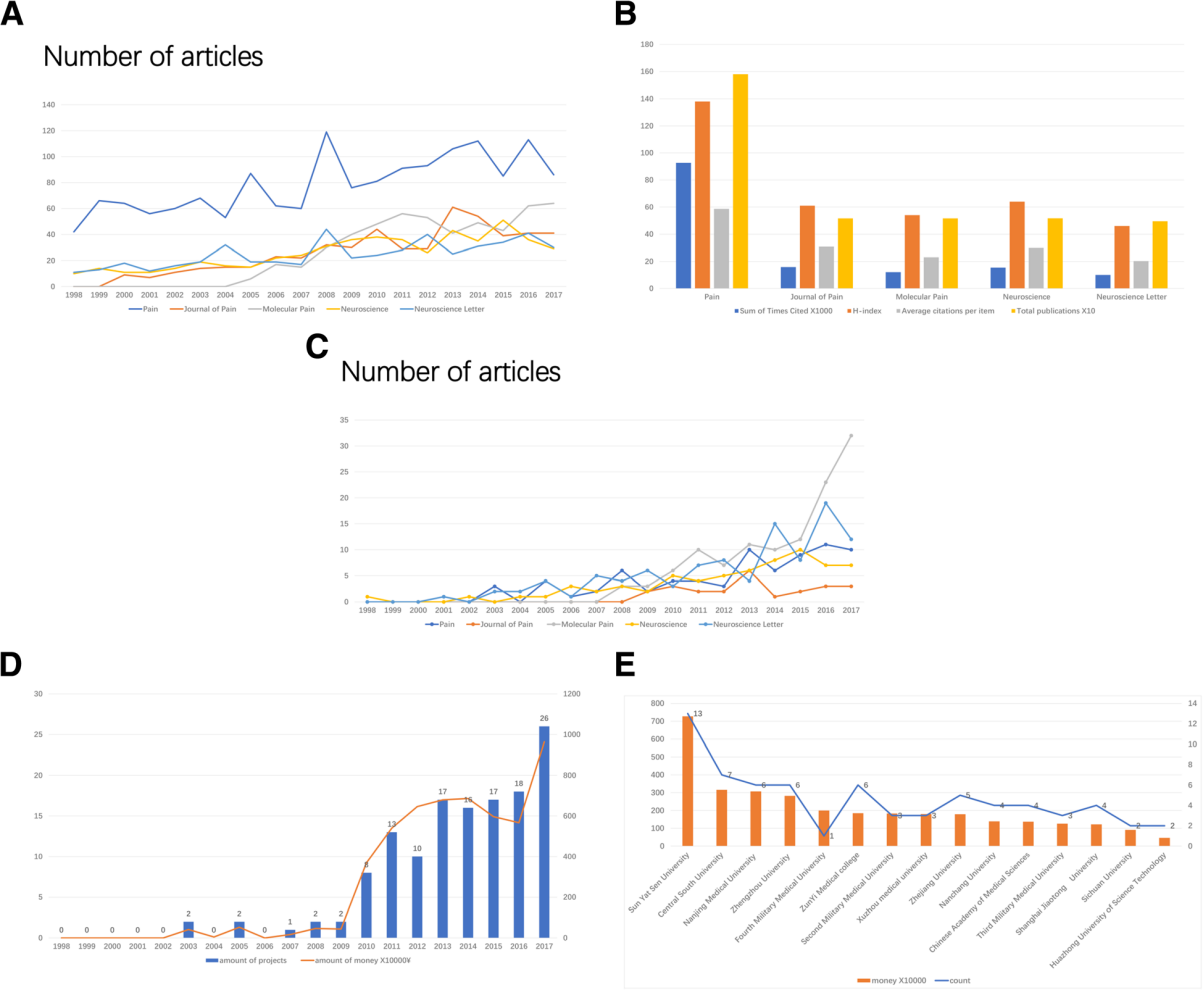
High contribution journals in neuropathic pain field and funds in China (**a**) The top5 high contribution journals in neuropathic pain field. (**b**) The number of articles for 5 main neuropathic pain journals in world. (**c**) The number of articles for 5 main neuropathic pain journals in China. (**d**) The time curves for NSFC funding in past 20 years. (**e**) NSFC funding of neuropathic pain research at different Chinese institutions

In neuropathic pain field, PAIN is the most contribution journal in the world with the largest number of publications and highest H-index. For China, Molecular Pain produced the largest number of paper, with 117 neuropathic pain papers, accounting for 4.59% of the total, followed by Neuroscience Letters (101, 3.962%) and Pain (76, 2.982%) (Figs. 3a-c and 4a-c).

In China, National Natural Science Foundation of China (NSFC) was the main Chinese funding organization, which reflected the scientific capability of research institutions. The fund supplied a total of 134 projects and 52.551 million RMB for neuropathic pain research in the past 20 years, with a strong growth trend (for amount of projects: R^2^ = 0.7958, *P* < 0.01; for money: R^2^ = 0.7706, *P* < 0.01), indicating that neuropathic pain field gradually gained official attention and support. Among them, Sun Yat-Sen University obtained 7.27 million RMB funding of NSFC, followed by Central South University (3.16 million RMB) and Nanjing Medical University (3.07 million RMB) (Figs. 3d-e and 4d-e).

### The 10 most-cited articles on neuropathic pain in world and China

The top10 articles are listed in Tables 1 and 2. The number of citations for the global top10 articles ranged from 1060 to 2147, while the most-cited paper in China only cited 504 times. The top10 articles in both world and China mainly published from 1999 to 2014, while none were published in the latest three years (2015–2017). In addition, compared that the global top10 papers are all reviews, the top10 most-cited papers included 5 articles, 4 reviews and 1 brief communication.

**Table 1:** The Top 10 most frequently cited articles on neuropathic pain in the world

Title	Author	Journal	Year	IF	Times cited
Neuroscience - Neuronal plasticity: Increasing the gain in pain	Woolf, CJ et al	SCIENCE	2000	41.058	2147
Persistent postsurgical pain: risk factors and prevention	Kehlet, H et al	LANCET	2006	53.254	1470
Core outcome measures for chronic pain clinical trials: IMMPACT recommendations	Dworkin, RH et al	PAIN	2005	5.559	1422
Neuropathic pain: aetiology, symptoms, mechanisms, and management	Woolf, CJ et al	LANCET	1999	53.254	1309
Neuropathic pain - Redefinition and a grading system for clinical and research purposes	Treede, R.-D et al	NEUROLOGY	2008	7.609	1247
Cellular and Molecular Mechanisms of Pain	Basbaum, Allan I et al	CELL	2009	31.398	1224
Central sensitization: Implications for the diagnosis and treatment of pain	Woolf, Clifford J.	PAIN	2011	5.559	1190
Monocyte Chemoattractant Protein-1 (MCP-1): An Overview	Deshmane, Satish L et al	JOURNAL OF INTERFERON AND CYTOKINE RESEARCH	2009	2.419	1138
Central Sensitization: A Generator of Pain Hypersensitivity by Central Neural Plasticity	Latremoliere, Alban et al	JOURNAL OF PAIN	2009	4.859	1107
Pharmacologic management of neuropathic pain: Evidence-based recommendations	Dworkin, Robert H et al	PAIN	2007	5.559	1060

**Table 2:** The Top 10 most frequently cited articles on neuropathic pain in China

Title	Author	Journal	Year	IF	Times cited
Cytokine mechanisms of central sensitization: Distinct and overlapping role of interleukin-1 beta, interleukin-6, and tumor necrosis factor-beta in regulating synaptic and neuronal activity in the superficial spinal cord	Kawasaki, Yasuhiko et al	JOURNAL OF NEUROSCIENCE	2008	5.970	504
Neural mechanism underlying acupuncture analgesia	Zhao, Zhi-Qi	PROGRESS IN NEUROBIOLOGY	2008	14.163	394
Acupuncture and endorphins	Han, JS	NEUROSCIENCE LETTERS	2004	2.159	360
Identification of gene expression profile of dorsal root ganglion in the rat peripheral axotomy model of neuropathic pain	Xiao, HS et al	PNAS	2002	9.504	342
Emerging targets in neuroinflammation-driven chronic pain	Ji, Ru-Rong et al	NATURE REVIEWS DRUG DISCOVERY	2014	50.167	196
Brain cannabinoid CB2 receptors modulate cocaine’s actions in mice	Xi, Zheng-Xiong et al	NATURE NEUROSCIENCE	2011	19.912	170
Down-regulation of mu-opioid receptors in rat and monkey dorsal root ganglion neurons and spinal cord after peripheral axotomy	Zhang, X et al	NEUROSCIENCE	1998	3.338	145
The role of tumor necrosis factor-alpha in the neuropathic pain induced by Lumbar 5 ventral root transection in rat	Xu, Ji-Tian et al	PAIN	2006	5.559	141
Emerging role of Toll-like receptors in the control of pain and itch	Liu, Tong et al	NEUROSCIENCE BULLETIN	2012	3.155	138
Spinal glial activation in a new rat model of bone cancer pain produced by prostate cancer cell inoculation of the tibia	Zhang, RX et al. Berman, BM; Lao, LX	PAIN	2005	5.559	132

## Discussion

In our studies, we completed a bibliometric study of the scientific publications in global neuropathic pain research and analyzed the China’s achievement and gap in neuropathic pain field in the last 2 decades, which helped us to comprehend the global scientific trend in neuropathic pain and the direction of the scientific and technological innovation in China. In the past 20 years, neuropathic pain research had made a continuous and rapid growth in number of papers at world level. Like its economic power, the USA held the first place in every category, with largest publications and citations and highest H-index. The scientific research and development are still unbalanced, and developed countries are leading the global neuropathic pain trend. China scored a tremendous achievement in neuropathic pain field. However, compared to articles published by other leading countries, the quality of China’s papers still needs to be improved. In terms of articles type, same as the world, Chinese researchers also focused more attention on basic research with more output. Accompanied by official attention and support, neuropathic pain related NSFC funding became more and more, which enhanced the development of China’ scientific research further.

Amazing achievement were acquired in global neuropathic pain research, with 320 papers in 1998 increasing to 2723 in 2017, which inspired those people suffered from pain with neuropathic characteristics. Based on these researches and outcomes, neuropathic pain was classified more and more accurate, and its several important contributory mechanisms included abnormal discharge in nociceptive nerves, peripheral and central sensitization, chronic and pathological activation of microglia, and impaired inhibitory modulation [6]. Current treatment still recommended antidepressants (tricyclic agents and serotonin-norepinephrine reuptake inhibitors) and anticonvulsants (gabapentin and pregabalin) as first-line treatments [4]. Individualized multidisciplinary patient care provided new direction for neuropathic pain, which may cause further advancements to neuropathic pain research in the future.

In the early decade, neuropathic pain research in China seemed to be faltering, but burst after 2010, reaching 223 articles in 2013, which ranked second in the world and was in accordance with NSFC funding assistance in 2010. The following reasons may explain this phenomenon: The scientific research power is based on economic strength. The world’s second largest economy boosted the neuropathic pain research in the past 20 years in China, which was also demonstrated by many bibliometric studies of other disciplines. The NSFC is a barometer of scientific research investments in China. Besides these funding of national level, most of the provinces would increase the supporting funding in neuropathic pain following national science and technology policy, such as provincial or municipal natural science fund key projects. It is because of these policy and funding that more and more talented person returned to China with advanced concept and techniques and promoted the international cooperation on neuropathic pain diagnosis and treatments. Additionally, the huge population base is a precious resource for clinical trial in neuropathic pain in China. That is why clinical trials and random controlled trials increased rapidly in the last ten years. However, completed clinical database and analysis system should be planned to unite and integrate these independent different hospital and massive amount of clinical resource.

Except for quantity increase, we should emphasize the quality of publications. For the citations frequency and h-index, there exists still a considerable gap between China and other developed countries. Compared to ourselves, our quality of neuropathic pain publications had improved. But it need more time to catch up other leading countries. At first, China should continue to introduce the top talents and leading professors to construct high quality neuropathic pain research platform and multi-center studies. In our bibliometric analysis, no Chinese institutions could rank into top20 contributing institutions. No experts, no institutions. Second, consideration that there is no top pain related journal from China, several international journals on neuropathic pain should be created in China so as to attract more submissions and spread academic perspective. Based on these situations, China still has great potential to grow in publications quality.

Obviously, there are some limitations in our analysis. Firstly, some research outcomes may involve in international collaboration with different countries. Our retrieved results of institutional affiliations could cause bias in the study. Second, on account of “neuropathic pain” as subject term, we may ignore some papers, such as those indexed with “chronic pain”. Third, some articles collection from WOS and PubMed database may be delayed so that citations and H-index exist flaw.

## Conclusion

At world level, neuropathic pain research had made impressive growth in volume during the last 20 years. The USA is still the leader of neuropathic pain research both in quality and quantity. In the past two decades, China gradually became a critical force on neuropathic pain research. However, compared with the publications quantity growth, there is still a considerable gap in research quality between China and other leading countries. Therefore, there is still a long way to go, China need take measure to complete high-quality neuropathic pain studies.

## Abbreviations

A&HCI: Arts & Humanities Citation Index
IF: impact factors
NSFC: National Natural Science Foundation of China
RCTs: randomized controlled trials
SCIE: Science Citation Index Expanded
SSCIE: Social Sciences Citation Index
WOS: Web of Science

## References

[1] van Hecke O, Austin SK, Khan RA, Smith BH, Torrance N (2014). Neuropathic pain in the general population: a systematic review of epidemiological studies. Pain.

[2] Guevara-López U, Covarrubias-Gómez A, García-Ramos G, Hernández-Jiménez S (2006). Practice guidelines for neuropathic pain management. Rev Investig Clin.

[3] Jensen TS, Baron R, Haanpää M (2011). A new definition of neuropathic pain. Pain.

[4] Gilron I, Baron R, Jensen T (2015). Neuropathic pain: principles of diagnosis and treatment. Mayo Clin Proc.

[5] Mayoral V, Pérez-Hernández C, Muro I, Leal A, Villoria J, Esquivias A (2018). Diagnostic accuracy of an identification tool for localized neuropathic pain based on the IASP criteria. Curr Med Res Opin.

[6] Cohen SP, Mao J (2014). Neuropathic pain: mechanisms and their clinical implications. BMJ.

[7] Teles AR, Ocay DD, Shebreen AB et al (2018) Evidence of impaired pain modulation in adolescents with idiopathic scoliosis and chronic back pain. Spine J 10.1016/j.spinee.2018.10.009 30343045

[8] Schou WS, Ashina S, Amin FM, Goadsby PJ, Ashina M (2017). Calcitonin gene-related peptide and pain: a systematic review. J Headache Pain.

[9] Truini A (2015). Trigeminal neuralgia. J Headache Pain.

[10] Xie G, Zhang K, Wood C, Hoeft A, Liu J, Fang X (2016). China's contribution to anesthesiology research: a 10-year survey of the literature. Anesth Analg.

[11] Ye B, Du TT, Xie T (2014). Scientific publications in respiratory journals from Chinese authors in various parts of North Asia: a 10-year survey of literature. BMJ Open.

[12] Rezaee ME, Johnson HA, Munarriz RM, Gross MS (2018) Bibliometric analysis of erectile dysfunction publications in urology and sexual medicine journals. J Sex Med 10.1016/j.jsxm.2018.08.004 30219665

[13] Zhu H, Yang X, Qin Q (2014). Report of China's innovation increase and research growth in radiation oncology. Chin J Cancer Res.

